# Changes in emotion regulation skills of school age children from the eyes of healthcare workers during the COVID‐19 pandemic in Turkey: A cross‐sectional study

**DOI:** 10.1002/hsr2.964

**Published:** 2022-11-30

**Authors:** Bedriye Tuğba Karaaslan, Gözde Akoğlu, Gonca Özyurt, Elif Oral

**Affiliations:** ^1^ Department of Child Development Faculty of Health Sciences, İzmir Katip Çelebi University İzmir Turkey; ^2^ Child Psychiatry Department Faculty of Medicine, İzmir Katip Çelebi University İzmir Turkey; ^3^ Psychiatry Department Faculty of Medicine, İzmir Katip Çelebi University İzmir Turkey

**Keywords:** children, COVID‐19 pandemic, emotion regulation, healthcare workers

## Abstract

**Background and Aims:**

While health workers were affected by the COVID‐19 pandemic on an individual and professional basis, their families and especially their children were directly or indirectly exposed to similar factors. This study aims to compare the emotion regulation competencies of school‐age children of healthcare workers in Turkey, before and during the COVID‐19 pandemic and to investigate their emotion regulation competencies during the pandemic in terms of care conditions, daily life activities and overall health.

**Methods:**

This study used the purposeful sampling technique and was designed as a cross‐sectional and relational survey study. To conduct this study, permission was received from the Ministry of Health Directorate‐General for Health Services, COVID‐19 Research Evaluation Commission, and İzmir Kâtip Çelebi University Non‐Interventional Clinical Research Ethics Committee (Date/Decision no: 04.03.2021/0090). The data‐collection process was carried out online between March and July, 2021. A total of 106 healthcare workers who serve in pandemic clinics or fields participated in this study. In addition to the information forms prepared by the researchers of this study, the Emotion Regulation Scale (ERS), which was adapted into Turkish, was used. One‐way analysis of variance for independent samples was used for the analysis of variables, and paired samples t‐test was used to compare emotion regulation competencies before and during the pandemic.

**Results:**

The children's total ERS scores increased during the pandemic (47.70 ± 8.35) when compared with their pre‐pandemic scores (44.86 ± 9.25), and furthermore this difference was found to be statistically significant (*p* = 0.000). The total mean ERS score of children with a healthcare worker parent increased significantly during the pandemic compared with the scores before the pandemic, which revealed that emotion regulation occurred at a lower level for these children.

**Conclusion:**

The effect of the COVID‐19 pandemic on the emotion regulation competencies of children whose parents are healthcare workers is evident.

## 1 INTRODUCTION

The new type of Coronavirus (COVID‐19) pandemic has unsettled the entire world over the past 2 years and has, primarily, confronted countries with a health crisis. Indeed, in the fight against the pandemic, healthcare workers have several primary concerns: access to the appropriate personal protection equipment, being contracted COVID‐19 on duty, transmitting the disease to their families and colleagues, inability to afford personal and family expenses in case of getting infected, care of their children due to the closure of schools, the failure to meet personal and family needs as working hours and overtime requests increase, and lack of communication and up‐to‐date information. In addition to these concerns, reasons such as psychological burden, psychosocial pressure, burnout syndrome, discrimination and labeling, physical and psychological abuse, and violence have resulted in healthcare workers being more affected by the pandemic.^1^, ^2^, ^3^

While healthcare workers are affected by the pandemic at an individual and professional base, their families and particularly their children are exposed to similar factors, either directly or indirectly. Reasons such as their children's lack of understanding regarding the isolation precautions, the closure of centers that can provide caring services for children, the decrease in time spent together, the diversification and increase of children's support needs during the distance‐education period, or the fear of parents' getting sick or losing them, has caused healthcare workers to go through a work‐family conflict and to experience more concerns regarding their children's health and development.^4^, ^5^, ^6^

The literature highlights that the pandemic has deeply affected people's lives across the whole world and that measures such as isolation, contact limitations, and economic lockdown essentially threaten the mental health of children and adolescents. Moreover, it is reported that the daily routines such as sleeping, nutrition, playing, amd so forth, and of the mental health of both parents and children have been negatively affected by the pandemic process. During the pandemic, anxiety, lack of peer interaction, and decrease in opportunities for emotion regulation (adjustment) were among the main concerns of experts.^5^

Emotion regulation is considered an essential source for children's development, particularly when facing difficulties, and several comparative studies have been conducted on this issue.^7^ In keeping with a perspective that conceptualizes development as the increasing diversification and hierarchical integration of biological and psychological systems, it is highlighted that children's emotion regulation competencies become more complicated and integrated during preschool and primary school years. Researchers underline the need to measure the emotion regulation competency in a more significant number of situations of increasing difference to better understand this complex development process.^8^

Many different studies have been undertaken to depict the situation in Turkey, on healthcare personnels' physical and psychological wellness during the pandemic, some of which are revisions or letters to the editor and some of which are original research papers.^4^, ^6^, ^9^, ^10^, ^11^, ^12^, ^13^, ^14^, ^15^ However, only a few studies that inform about changes in children's physical, psychological, and developmental states were encountered in the related Turkish literature.^16^, ^17^, ^18^, ^19^ It is of critical importance to know the implicit consequences of the pandemic on children to support children and families in a more qualified way. In this regard, considering the fact that emotion regulation is the leading dimension of psychological health, this study investigated the emotion regulation competency of children of healthcare workers in terms of various variables during the COVID‐19 pandemic.

## 2 METHODS

### 2.1 Study design and duration

The study was designed online as a cross‐sectional and relational survey study using the sampling technique for research purposes. Considering the pandemic conditions, all data were collected online between March and July 2021.

### 2.2 Participants

The sample size was measured using G*power 3.1.9.2 with *α* = 0.05, power (1−*β*) = 0.80 and the effect size is 0.3 and the sample size obtained was 90.^20^

The criteria for inclusion in the study are being a health worker working in public or private health institutions, having worked or working in pandemic clinics and the field, having children in the school age group (6−12 years old), and volunteering to participate in the study. However, the exclusion criteria from the study were not working in pandemic clinics and the field and having a child out of school age. The sample included 106 healthcare professionals who gave consent to participate in the study, worked in pandemic clinics/field and had school‐age children, and information was obtained from healthcare professionals about the emotional regulation of school‐age children before and during the pandemic.

### 2.3 Data collection tools

A Patient Demographic Information Form, the Information Form for Parents' Working Conditions, and Information Form for School‐Age Children and The Emotion Regulation Scale (ERS) were used to gather data. An informed consent form was presented on the first page of each form and scale used in this study. Informed consent text with instructions for each form and scale was presented on the first page of the online form, and it was possible for the parents of health professionals who gave consent to fill out the form. Online written informed consent was obtained from all healthcare worker parents participating in this study.

The following data‐collection tools were used in the present study: Demographic Information Form for Parent was used to gather information on the demographics of healthcare worker parents; the Information Form for Parent's Working Conditions was used to collect information on the working conditions of the parents, and the Information Form for Parents' School‐Age Children of Parent was used to gather information regarding the care conditions and daily life activities of parents of children aged 6–12 years. The researchers of the present study created these forms for use within the scope of this study.

The ERS was used to determine the children's emotion regulation competencies before and during the pandemic.

#### 2.3.1 ERS

The Scale, originally titled Emotion Regulation Checklist, was developed by Shields and Cicchetti, was used to determine the children's emotion regulation competencies before and during the pandemic.^8^, ^21^ Kapçı et al. adapted this Scale into Turkish, and permission was obtained to use the Scale for the present study.^21^ The Scale can be filled out by a child's mother, father, and teacher, as well as any adult who knows the child. The Scale comprises 24 4‐point Likert‐type items indicating never “1,” occasionally “2,” frequently “3,” and almost always “4.” It takes approximately 10 min to fill out the Scale. The Scale consists of items that evaluate the intensity, instability, and value (positivity or negativity) of emotions and the average appropriateness of the emotional statement. Shields and Cicchetti, as a result of their analysis, determined that the Scale has two factors, Instability/Negativity, and Emotion Regulation: The Instability/Negativity factor involving factors related to mood oscillations, namely acting heatedly, emotional intensity, and the regulation of positive emotions; the Emotion Regulation factor involving items related to adaptive emotion regulation, such as understanding emotions, empathy, and equanimity.^8^, ^21^ Kapçı et al. adapted the Scale into Turkish with a study conducted with 284 children in 2009. The correlation between the evaluations made by the mothers and fathers (*r* = 0.81), test−retest reliability (*r* = 0.90), and internal consistency (*α* = 0.84) determined that the Scale is highly reliable in its evaluation. Consequently, the researchers proved that the Scale could be used to evaluate the emotion regulation competencies of 6–12‐year‐old children.^21^ A high total score obtained from the scale indicates that the child's emotion regulation is at low level. A high first factor (Instability/Negativity) score indicates that the child is unable to regulate emotions, and a high second‐factor score (Emotion Regulation) indicates that the child has a good level of emotion regulation.^21^

### 2.4 Data analysis

SPSS (Statistical Package for the Social Sciences) 26.0 (IBM Corp.) was used for data analysis. To check the accordance of variables with the normal distribution of data, analytic methods (Kolmogorov–Smirnov/Shapiro–Wilk tests), as well as kurtosis and skewness coefficients were used (±1). One‐way analysis of variance for Independent Samples was used for the analysis of the variables that were found to be normally distributed, and related samples *t*‐test was used to compare the children's emotion regulation competencies before and during the pandemic. Throughout the study, values *p* < 0.05 were considered to be statistically significant.

## 3 RESULTS

As a result of post hoc analysis, the power was determined as 86% for 106 participants.

The sociodemographic information of the participants and their children is presented in Table 1. Participants' mean age was 40.69 ± 4.06 years. Of the parents who filled out the form, 89 (84%) were females, and 17 (16%) were males. 103 (97.2%) of the parents had a university degree or higher level of education, and only 3 (2.8%) were high‐school graduates. Overall, 40 (37.7%) were specialist doctors, 21 (19.8%) were dentists, 20 (18.9%) were nurses, 14 (13.2%) were support health personnel, and 11 (10.4%) were other healthcare workers (e.g., civil servants and technicians). Considering the working conditions of these parents and healthcare workers over the past month, 70 (66%) reported that they had worked according to their regular shift system; 15 (14.1%) reported that they worked according to their flexible shift system, in addition to head duty; 12 (11.4%) reported that they worked in COVID‐19 services and patient admissions to these services, and 9 (8.5%) reported that they worked with flexible working hours. Of the children in the sample, their mean age was 8.99 ± 1.98; 49 (46.2%) were females, and 57 (53.8%) were males. Overall, 3 (2.8%) of the children had not started school due to the pandemic, 10 (9.4%) were in kindergarten, 62 (58.5%) were in primary school, and 31 (29.2%) were in secondary school.

**Table 1:** Demographic characteristics of the study population (*n* = 106)

Details of the parents	Frequency (*n*)	Percentage (%)
Age (Mean ± SD)	40.69 ± 4.06	
Gender		
Male	17	16
Female	89	84
Education		
High school	3	2.8
University degree	103	97.2
Profession		
Specialist doctors	40	37.7
Dentists	21	19.8
Nurses	20	18.9
Supporting staff	14	13.2
Others	11	10.4
Working condition		
Regular shift	70	66
Flexible shift	15	14.1
Other	21	19.9
Details of the children		
Age (Mean ± SD)	8.99 ± 1.98	
Gender		
Male	57	53.8
Female	49	46.2
Education		
Not yet started school	3	2.8
Kindergarten	10	9.4
Primary	62	58.5
Secondary	31	29.2

Abbreviation: SD, standard deviation.

According to the parents' opinions regarding the children's emotion regulation competencies, 62 (58.5%) of the parents stated that there had been no change in their children's emotion regulation competencies during the pandemic when compared with their prepandemic competencies. In comparison, 44 (41.5%) stated that there had been a negative change. Results on the comparison of the children's mean ERS scores before and during the pandemic are presented in Table 2. The results showed that the children's mean ERS Instability/Negativity subscale scores significantly increased during the pandemic (29.25 ± 6.01) when compared with their prepandemic scores (27.32 ± 6.35) and that furthermore, the difference between their mean scores before and during the pandemic was significant (*p* = 0.000). Similarly, children's mean ERS Emotion Regulation subscale scores were found to significantly increase during the pandemic (18.44 ± 3.84) when compared with their prepandemic scores (17.54 ± 4.11), and, furthermore, this difference was found to be statistically significant (*p* = 0.001). Overall, the children's total ERS scores increased during the pandemic (47.70 ± 8.35) when compared with their pre‐pandemic scores (44.86 ± 9.25), and furthermore this difference was found to be statistically significant (*p* = 0.000).

**Table 2:** Comparison of the children's ERS scores before and during the pandemic (*n* = 106)

Children's ERS scores	Before pandemic	During pandemic	Cohen's *d*	*p* Value
	Mean (SD)	Mean (SD)		
Instability/Negativity Subscale	27.3 (6.35)	29.25 (6.01)	0.31	0.000*
Emotion Regulation Subscale	17.54 (4.11)	18.44 (3.84)	0.22	0.001*
ERS Total Score	44.86 (9.25)	47.70 (8.35)	0.32	0.000*

Abbreviations: ERS, emotion regulation score; SD, standard deviation.

* *p* < 0.05.

Table 3 presents the descriptive statistics on the children's general care conditions and daily life activities during the pandemic and the results on the comparison of their scores on the ERS in line with these findings. As per the children's score on the ERS, there were significant negative changes in the playing habits, routine nutrition intake and self‐care during the pandemic as shown in Table 3. In addition, although this is not significant, more than half of the parents (55.2%) reported negative changes in their children's sleep routine. Finally, when the children's mean ERS scores were compared according to whether or not they have been apart from their parents and their mode of staying at home during the pandemic, the difference between these groups was not found to be significant.

**Table 3:** Comparison of the children's scores on the ERS according to their care conditions and daily‐life activities during the pandemic (*n* = 106)

		*n*	%	Mean ± SD	*p* Value	*η* ^2^
Parent's being apart from child	I have never been apart.	57	53.8	47.00 ± 8.75	0.356	0.41
	I have been apart.	49	46.2	48.51 ± 7.87		
Way of Staying at Home	S/he stayed alone at home.	21	19.8	46.90 ± 4.73	0.629	0.37
	An adult gave care.	85	80.2	47.89 ± 9.04		
Sleep Routine	No, there has been no change.	47	44.8	46.59 ± 8.18	0.901	0.46
	Yes, there has been a negative change.	58	55.2	48.72 ± 8.45		
Nutrition Routine	No, there has been no change.	49	46.2	45.57 ± 8.24	0.043*	0.54
	Yes, there has been a positive change.	9	8.5	48.11 ± 8.40		
	Yes, there has been a negative change.	48	45.3	49.79 ± 8.07		
Self‐care Skills	No, there has been no change.	64	60.4	46.57 ± 7.70	0.000*	0.51
	Yes, there has been a positive change.	13	12.3	42.53 ± 4.11		
	Yes, there has been a negative change.	29	27.4	52.48 ± 9.04		
Playing Habits	No, there has been no change.	21	19.8	43.00 ± 6.21	0.001*	0.48
	Yes, there has been a positive change.	2	1.9	36.50 ± 3.53		
	Yes, there has been a negative change.	83	78.3	49.15 ± 8.30		

Abbreviation: SD, standard deviation.

* *p* < 0.05.

Descriptive statistics on the children's general state of health and development during the pandemic, and the results of the comparison of their ERS scores in line with these findings, are presented in Table 4. When the children's mean ERS scores were compared with their contracting COVID‐19, chronic disease, or special need, no significant difference was found between the groups.

**Table 4:** Comparison of the children's ERS scores according to the effect of the pandemic process on their general health and development (*n* = 106)

		*n*	%	Mean ± SD	*p* Value	*η* ^2^
General state of health	S/he has been COVID‐19 positive.	7	6.6	46.57 ± 5.74	0.461	0.18
	Health follow‐ups before the pandemic have been slowed.	12	11.3	50.08 ± 6.51		
	S/he has developed new health issues independent of COVID‐19.	5	4.7	51.80 ± 6.57		
	There has been no issue affecting their health.	82	77.4	47.19 ± 8.82		
Having a chronic disease or special need	S/he has no chronic diseases or special needs.	89	84.0	47.17 ± 7.86	0.145	0.22
	S/he has a chronic disease or at least one special need in the fields of cognitive/mental/physical/language/vision/hearing.	17	16.0	50.41 ± 10.43		

Abbreviation: SD, standard deviation.

## 4 DISCUSSION

When the children's results on the ERS, which are based on the parents' statements, were investigated, the means of the scores obtained on the Instability/Negativity subscale were found to significantly increase during the pandemic when compared with the children's prepandemic scores. This increase indicated a negative change, especially in the ability of children to regulate their positive emotions. Comparatively, the children's mean scores on the emotion regulation subscale during the pandemic were found to be significantly higher than their prepandemic scores, indicating a positive change in their emotion regulation competency. It was also found that the children's total mean ERS scores significantly increased during the pandemic compared with their prepandemic scores and that, therefore, the emotion regulation has occurred at a lower level; however, oscillations in their moods, emotional intensity, and difficulties to improve positive emotions were also found due to the two‐factor structure of the scale, and, furthermore, emotion regulation functions, such as understanding emotions, empathy, and equanimity, among the children were found to improve. This result concerning the children's total mean scores mainly points out that the pandemic has negatively affected their emotion regulation competency.

Concerning the parents' subjective opinions regarding their children's emotion regulation competencies, 58.5% stated that there has been no change in their children's emotion regulation competency due to the pandemic. Those parents (41.5%) who stated that there has been a negative change in their child's emotion regulation competency responded to the open‐ended question that asked how this change occurred by saying that their child was “angrier, more anxious, more resistant to rules, more demanding, more fragile, more antisocial, and more stuck on screen” and stated their concerns regarding this issue.

A study conducted in Brazil investigated parents' perceptions regarding the emotion regulations of their children aged 5–12 years and the comparison of the perceptions before and during the lockdown in the current pandemic. The results, which are in line with those of the present study, showed that there is a significant increase in children's emotion regulation, as perceived by their parents during the lockdown, in regard to both their total scores and their mean scores for the emotion regulation subscale. Comparatively, and in contrast to our findings, a significant decrease was detected in their mean Instability/Negativity subscale scores. However, this decrease was highlighted as a wanted state for the adjustment of positive emotions. As a result of that study conducted in Brazil, it was revealed that, despite all challenges associated with the lockdown, children's emotion regulation competencies increased during this lockdown process.^7^ From a developmental perspective, the middle‐childhood period, which includes emotion regulation competencies, is a period in which emotion regulation competency is enhanced and those skills needed to positively reevaluate emotional awareness and situations that distract a child's attention or that create disappointment are improved.^22^ The results of both aforementioned studies point out that children of this age group have some difficulties regulating their emotions and that they put effort into improving their emotion regulation strategies during the pandemic when compared to other development stages.

When the children's mean ERS scores were compared according to whether or not they have been apart from their parents and their mode of staying at home during the pandemic, the difference between these groups was not found to be significant. In a different study conducted in Turkey with 457 healthcare workers who have children aged 6–16 years, it was determined that children of those healthcare workers who had experienced the pandemic in all aspects had less physical contact with their parents and, therefore, constitute a notable risk group since they experienced those measures taken against the pandemic more profoundly.^23^ Even if children are not separated from their parents, the lack of physical contact with their parents is reported as a risk for children. Nevertheless, being apart from their parents or staying at home alone while being unready for the experience are challenging elements for both psychological and safety reasons.

When parents who worked as healthcare workers were asked whether or not there has been a negative change in their children's daily life activities, they reported significant negative changes in their playing habits (78.3%), followed by nutrition routine (45.3%) and self‐care skills (27.4%). Moreover, 55.2% of parents reported that there had been negative changes to their children's sleeping routines. However, the difference between these groups was not significant. A different study that investigated the anxiety levels and sleeping problems of children of healthcare workers who had COVID‐19 reported that some anxiety problems, independent of COVID‐19, had been observed in these children; the same study also reported that parents' being apart from home during the pandemic is a predictor of children's sleeping disorders.^19^ This study also determined that there is a positive correlation between the anxiety of healthcare workers and their children; this correlation may explain the negative changes observed in children's daily life activities in the present study.^18^

In a study that compared the anxiety and depression levels of children (aged 9–17 years) of healthcare workers with those of nonhealthcare workers' children (also aged 9–17 years) in the first year of the pandemic, no significant difference was detected between the two groups in terms of their levels of depression levels. However, the study also found a significant difference between the two groups of children regarding anxiety levels, with the children whose parents were healthcare workers experiencing more anxiety. These findings are critical because they show that children whose parents work in highly risky settings are at greater risk compared with other children.^16^

During the pandemic, healthcare workers who assumed the responsibility of protecting and curing society as well as themselves and their families needed help in supporting their children. According to a study on the telehealth system that was established to provide psychiatric assistance to healthcare workers in Turkey, it was determined that one‐quarter of the users of the system asked for support in taking care of their children.^17^ The specialists in the aforementioned study stated that they were particularly concerned that single parents and parents who are at the front lines of the pandemic, such as healthcare workers, might experience more trouble regarding their children's health and development. Indeed, the literature highlights that measures taken against the pandemic, such as pausing education, implementing distance education, or social distancing, cause long‐term inaction, which indirectly disturbs children's sleeping and nutrition routines, causes the adaptation of a more sedentary lifestyle, causes changes in playing habits due to longer periods of screen use, and that all these factors may combine to cause childhood metabolic diseases.^24^, ^25^ Also through a mental perspective, various neuropsychiatric symptoms, such as stress, impatience, and uneasiness, are reported as potentially increasing.^24^ In a study conducted in Italy, it was stated that more than 30% of the adults and children in the pandemic area were at greater risk of post‐traumatic stress disorder and that this rate is higher for healthcare workers directly involved in COVID‐19 care and their children.^26^ Parents who could work from home experienced pandemic‐based stress and some difficulties in providing full‐time attentive childcare. In addition, due to opportunities missed concerning education and interaction with other children, the inability to present stimuli in a desired quantity and quality that support developmental and play environments has caused parents to raise concerns regarding their children's development and behaviors. Indeed, based on this point, a comparison of developmental evaluation data from comprehensive longitudinal studies in the literature conducted in the last decade (2011−2019), and studies in the literature conducted between 2020 and 2021 was conducted; these studies used data concerning the follow‐ups of 672 children aged 3 months to 3 years without term or developmental problems. This comparison determined that the verbal, motor, and cognitive performance of children born during the pandemic were significantly lower than those of children born before the pandemic. These results highlight that the environmental changes related to the COVID‐19 pandemic significantly and negatively affect children's development, even in the absence of severe acute respiratory syndrome coronavirus 2 infection and COVID‐19 disease.^27^ In line with the results of this study, parents in our study also stated that they have some concerns regarding their children's academic and social development. In addition, 85.8% of the parents in the present study indicated that they developed concerns regarding their children's development during the pandemic, with the most frequent domains being, respectively, social‐emotional, academic, “increased screen time,” and health.

When the children's mean ERS scores were compared with their contracting COVID‐19, chronic disease, or special need, no significant difference was found between the groups. Nevertheless, the pandemic process is thought to affect all children in various ways, whether or not they have contracted COVID‐19 and whether or not they have a special need. Changes in daily routines, interruptions in educational/special educational services, and changes in domestic life during the pandemic have challenged all children and families.^28^ Since children with a chronic disease or a special need constituted only a limited portion of our study sample (16%), the reflection of these challenged on the emotion regulation competency might be greatly limited.

### 4.1 Implications

This study is valuable as it supports studies that emphasize that the children of healthcare worker parents may be at risk during the COVID‐19 pandemic period. It also reveals that in this context, emotion regulation competence can be considered as an indicator, especially for school‐age children. The COVID‐19 pandemic has caused healthcare workers to face new challenges in balancing workload and caregiving in Turkey, as in many other countries.

### 4.2 Limitations of the study

The adaptation of the ERS used by our study covers the 6–12‐year age interval. Therefore, it is a limitation of our study not to include the preschool and adolescent groups. Another limitation of the study is that it was not possible to conduct direct, face‐to‐face meetings with children due to the pandemic. Despite these limitations, the data collected by the present study are nevertheless thought to be helpful in terms of being acquainted with the difficulties healthcare workers and their children face, and in planning protective, preventive, or supportive services during biological disasters such as pandemics.

## 5 CONCLUSION

In conclusion, the effect of the COVID‐19 pandemic on children with a healthcare worker parent is evident, and this interaction will continue until the end of the pandemic. In exceptional situations like pandemics, the need to support children, especially those with healthcare worker parents, and their parents and caregivers should be given as much focus as is possible; this also applies to those with disadvantaged situations, such as having special needs, experiencing domestic violence, living in institutional care, having a low‐socioeconomic status, and having immigrant status. In this regard, it is of critical importance to plan future studies that can analyze and propound the effect of policies executed for managing the pandemic on children's development and mental health, evaluate the effects of measures that are to be taken, and determine the strength of each effect to be better prepared for any potential risks.

## AUTHOR CONTRIBUTIONS

**Bedriye Tuğba Karaaslan**: Conceptualization; data curation; formal analysis; methodology; writing – original draft; writing – review and editing. **Gözde Akoğlu**: Conceptualization; formal analysis; methodology; writing – review and editing. **Gonca Özyurt**: Methodology; writing – review and editing. **Elif Oral**: Writing – review and editing.

## CONFLICT OF INTEREST

The authors declare no conflict of interest.

## ETHICS STATEMENT

Before starting the implementation of the study, necessary permissions were received from the COVID‐19 Research Evaluation Commission of Directorate General for Health Services, Ministry of Health and İzmir Katip Çelebi University Non‐Interventional Clinical Research Ethics Committee (Date/Decision no: 04.03.2021/0090).

## TRANSPARENCY STATEMENT

The lead author Bedriye Tuğba Karaaslan affirms that this manuscript is an honest, accurate, and transparent account of the study being reported; that no important aspects of the study have been omitted; and that any discrepancies from the study as planned (and, if relevant, registered) have been explained.

## Data Availability

The data that support the findings of this study are available on request from the corresponding author. The data are not publicly available due to privacy or ethical restrictions. All authors have read and approved the final version of the manuscript. Bedriye Tugba Karaaslan had full access to all of the data in this study and takes complete responsibility for the integrity of the data and the accuracy of the data analysis.
